# Duchenne Muscular Dystrophy: Contemporary Therapeutic Options and Real-World Challenges in Treatment Selection

**DOI:** 10.3390/muscles5010021

**Published:** 2026-03-12

**Authors:** Maria Tozzo Pesco, Gülru Zeynep Öztürk, Shivkumar C. Bhadola, Stephen M. Chrzanowski, Liubov V. Gushchina, Eleonora S. D’Ambrosio

**Affiliations:** 1 Department of Genetic and Cellular Medicine, UMass Chan Medical School, Worcester, MA 01655, USA; eleonora.dambrosio@umassmed.edu; 2 School of Medicine, Ankara University, Ankara 06230, Turkey; gulruzeynepozturk@gmail.com; 3 Department of Neurology, UMass Chan Medical School, Worcester, MA 01655, USA; shivkumar.bhadola@umassmemorial.org (S.C.B.); stephen.chrzanowski@umassmed.edu (S.M.C.); 4 Department of Pediatrics, UMass Chan Medical School, Worcester, MA 01655, USA; 5 The Jerry R. Mendell Center for Gene Therapy, Nationwide Children’s Hospital, Columbus, OH 43215, USA; liubov.gushchina@nationwidechildrens.org; 6 Department of Pediatrics, The Ohio State University, Columbus, OH 43205, USA

**Keywords:** Duchenne muscular dystrophy, dystrophin, gene therapy, neuromuscular disease, micro-dystrophin, adeno-associated virus, corticosteroids

## Abstract

Duchenne muscular dystrophy (DMD) is a severe X-linked neuromuscular disorder caused by loss-of-function mutations in the dystrophin gene, leading to progressive muscle degeneration, motor decline, respiratory compromise, and cardiomyopathy. Diagnosis typically occurs in early childhood following recognition of motor delays, markedly elevated creatine kinase, and confirmatory genetic testing. Over the past decade, the therapeutic landscape for DMD has expanded substantially, evolving from exclusively supportive care to patient-centric multifaceted treatment paradigms, including corticosteroids, mutation-specific therapies, small molecule disease-modifying approaches, and gene replacement strategies. Despite these advances, no currently available therapy restores full-length dystrophin or completely halts disease progression. This review provides a clinically oriented comprehensive overview of currently Food and Drug Administration (FDA)-approved medications for DMD, with particular emphasis on corticosteroids, exon-skipping therapies, nonsense mutation readthrough agents, recently approved gene therapy, and select ongoing gene therapy trials. We summarize mechanisms of action, clinical efficacy, safety considerations, regulatory status, and highlight the challenges of integrating these therapies into longitudinal care. Through illustrative clinical vignettes, we highlight the real-world complexity of treatment selection, shared decision-making, and longitudinal care planning in contemporary DMD management.

## 1. Introduction

Duchenne muscular dystrophy (DMD) is a rare, X-linked recessive, fatal, degenerative disease of the muscle caused by pathogenic variants in the *DMD* gene, with an estimated prevalence of 1 in 5000 live male births [1]. The *DMD* gene (OMIM 300377) encodes dystrophin, a 427 kDa cytoskeletal protein essential for maintaining sarcolemmal integrity during contraction and co-localizing a number of other proteins to the sarcolemmal membrane. In the absence of dystrophin, muscle fibers are highly vulnerable to contraction-induced injury, resulting in repeated cycles of degeneration and incomplete regeneration. Over time, muscle’s regenerative capacity diminishes, and tissue is progressively and irreversibly replaced by adipose and fibrotic tissue.

Pathologic changes begin in utero, which explains why boys with DMD are already born with markedly elevated creatine kinase (CK) levels [2]. Clinical severity varies widely; most infants demonstrate early developmental delay and achieve gross motor milestones later than peers. Calf hypertrophy is among the first clinically noticeable signs, followed by toe walking, difficulty climbing stairs, and frequent falls. Historically, loss of ambulation occurred in early adolescence, though advances in medical and supportive care have delayed this milestone for many patients into their mid-to-late teenage years.

Progressive involvement of respiratory and cardiac muscle remains the major determinant of long-term morbidity and mortality. Respiratory muscle weakness leads to restrictive lung disease and hypoventilation, while cardiomyopathy results from progressive myocardial fibrosis and ventricular dysfunction. Despite significant improvements in survival, cardiorespiratory complications remain the leading causes of death in individuals with DMD.

## 2. Methods

We conducted a narrative review of FDA-approved therapies and late-stage investigational treatments for Duchenne muscular dystrophy as of December 2025. Literature sources included PubMed-indexed publications, regulatory documents, clinical trial registries (clinicaltrials.gov), and publicly available prescribing information. Search terms included Duchenne muscular dystrophy, corticosteroids, prednisone, deflazacort, vamorolone, givinostat, exon skipping, antisense oligonucleotides, fast-twitch myosin inhibitors, satellite cell modulation, and gene therapy. This review emphasizes clinical trial data, safety profiles, and real-world considerations relevant to therapeutic decision-making. Fictionalized clinical scenarios are included to illustrate common treatment dilemmas encountered in clinical practice.

## 3. Diagnosis

Until the anticipated widespread implementation of newborn screening (NBS) for DMD, diagnosis typically takes place in early childhood years after caregivers or clinicians recognize delayed motor milestones or suggestive signs of proximal weakness, such as frequent falling, difficulty climbing stairs, toe walking, proximal weakness, a positive Gowers’ sign, or hypertrophic calves. Serum analysis almost always reveals elevated CK and transaminases (AST and ALT) with normal gamma glutamyl transferase [3]. It is noted that the elevated transaminases are resultant of injury to the muscle rather than hepatic etiology, often leading to unnecessary GI workups. These findings prompt referral to Child Neurology and ultimately confirmatory genetic testing. Though historically performed, muscle biopsies remain reserved for atypical phenotypes or when genetic results fail to fully explain the clinical phenotype [4]. Approximately 70% of patients harbor single-exon or multi-exon deletions or duplications in the dystrophin *DMD*, making deletion/duplication testing the first-line confirmatory test [3,4,5]. If negative, sequencing should be performed to detect other mutations, including point mutations, small deletions, or small insertions [3].

Newborn screening for DMD is an emerging diagnostic strategy with growing implementation within the United States. As of December 2025, the Recommended Uniform Screening Program (RUSP) formally recommended that all states screen for DMD, though execution and implementation remain up to individual states. Early identification through newborn screening enables pre-symptomatic diagnosis before symptom onset, facilitates timely initiation of disease-modifying therapies, and allows for early cardiac and respiratory surveillance. As newborn screening expands, clinicians must adapt counseling, monitoring, and treatment paradigms to pre-symptomatic populations, where long-term benefits and risks of early intervention remain under active investigation.

## 4. Epidemiology

The prevalence of DMD is less than 10 cases per 100,000 males, with an incidence of approximately 1 in 5000 male births [1]. Survival has improved substantially over recent decades, largely due to advances in respiratory support, cardiac management, and multidisciplinary care [4]. In a French study, the median life expectancy for individuals born before 1970 was 25.77 years, whereas for those born after 1970 was 40.95 years, underscoring the impact of modern supportive care on long-term outcomes [6].

## 5. Treatments

Contemporary management of DMD increasingly requires individualized risk–benefit assessment rather than algorithmic treatment selection. Standard care includes long-term corticosteroids, proactive cardiac and respiratory management, anticipation and prevention of the endocrinologic side effects of long-term steroids, orthopedic surveillance, physical and occupational therapy, and psychosocial support. Multidisciplinary neuromuscular care remains essential. Recently, disease-modifying therapies, including exon skipping, gene replacement approaches, and mutation-agnostic agents have expanded available treatment options. While emerging therapies offer meaningful advances, none restore full-length dystrophin or eliminate disease progression. As such, treatment decisions must integrate disease stage, genotype, comorbidities, family priorities, logistical burden, regulatory constraints, and uncertainty regarding long-term efficacy.

### 5.1. Glucocorticoids

Glucocorticoids remain the foundational therapy for DMD and are recommended when tolerated, even after loss of ambulation. Long-term corticosteroid use delays the loss of ambulation, preserves upper limb and respiratory function, and reduces the need for scoliosis surgery. However, these benefits must be weighed against well-documented adverse effects, and no glucocorticoid regimen fully prevents disease progression [3]. Recommended starting doses are 0.75 mg/kg/day for prednisone, 0.9 mg/kg/day for deflazacort [3], and 6 mg/kg/day for vamorolone [7].

Historically, prednisone and deflazacort have remained the mainstays of glucocorticoid therapy, until the advent of vamorolone in 2023. Both prednisone and deflazacort demonstrate clinical benefit, without clear preference for one over the other. Daily dosing is associated with more adverse effects, and recent studies suggest that twice-weekly regimens may be effective. Weekend dosing regimens (e.g., administering prednisone at 5 mg/kg on two consecutive days during the weekend) have been studied and demonstrated equivalent efficacy to daily prednisone in terms of preserving muscle strength and function over 12 months [8]. A randomized, double-blind trial of 64 boys with DMD weekend prednisone dosing (10 mg/kg/week given on two consecutive days) showed equivalence to daily prednisone (0.75 mg/kg/day) by way of upper and lower extremity quantitative muscle testing over 12 months. Overall side effect profiles, including height, weight, bone density, cataract formation, blood pressure, and behavior, did not differ significantly between the two regimens at 12 months [8].

In 2023, vamorolone, an oral selective dissociative corticosteroid approved for patients aged ≥2 years, was FDA approved [7]. Vamorolone acts overall on the same receptors as traditional corticosteroids but with altered activity at the glucocorticoid receptor (GR) and mineralocorticoid receptor (MR), causing it to be a glucocorticoid agonist and mineralocorticoid antagonist, and as such, may reduce some steroid-associated toxicities [7]. Vamorolone is available as an oral suspension, and the recommended dose is 6 mg/kg/day, taken once daily with a meal. Some patients may respond to a 2 mg/kg/day dose, which can be titrated as needed based on tolerability [7]. In patients with mild to moderate hepatic impairment, the recommended dose is 2 mg/kg/day once daily, adjusted downward if necessary [7]. For patients receiving a concomitant CYP3A4 inhibitor, the recommended dose is 4 mg/kg/day, once daily, and can also be titrated as needed. Short-term studies demonstrated improvements in functional outcomes such as the six-minute walk test and North Star Ambulatory Assessment relative to untreated cohorts, though direct comparative superiority over traditional corticosteroids has not been definitively established. The discontinuation of vamorolone should be achieved through gradual tapering. A study by C. Pascual-Morena et al. compared patients treated with vamorolone at 2 mg/kg/day and 6 mg/kg/day. At 24 weeks, the 6 min walk test (6MWT) increased by approximately 30 m and 29–45 m, respectively, while the North Star Ambulatory Assessment (NSAA) improved by 2.5–2.9 points, showing slightly better outcomes compared with untreated cohorts [9]. Based on these findings, vamorolone appears to be a viable alternative to traditional glucocorticoids, with a potentially lower incidence of adverse events; however, further studies are needed to confirm these observations. Long-term exposure to vamorolone may still be associated with side effects such as immunosuppression, decreased bone mineral density, secondary adrenal insufficiency, Cushing’s syndrome, hyperglycemia, behavioral and mood disturbances, and impaired growth and development [7]. The initial vamorolone dose after long-term corticosteroid use should be 6 mg/kg/day [7].

All glucocorticoid regimens require systematic monitoring and the treatment of adverse effects, including adrenal suppression, impaired linear growth, decreased bone mineral density, metabolic complications, delayed puberty, immunosuppression, and behavioral or mood disturbances [4]. Stress dose hydrocortisone is recommended for adrenal support at 50 mg for children < 2 years and 100 mg for older children and adults, as per the PJ Nicholoff Steroid Protocol for Duchenne and Becker muscular dystrophy and adrenal suppression recommends [10]. While vamorolone may reduce certain glucocorticoid-associated toxicities, long-term exposure remains associated with clinically meaningful steroid-related risks, underscoring the importance of ongoing surveillance regardless of agent selection. Importantly, vamorolone cannot be used as a stress dose steroid and requires special considerations for stress dosing.

### 5.2. Givinostat

Givinostat is an oral, non-steroidal histone deacetylase inhibitor, approved for patients aged ≥6 years with DMD [1]. Rather than restoring dystrophin, givinostat targets downstream pathogenic pathways, including inflammation and fibrosis, with the goal of slowing muscle degeneration. This ultimately results in slowed disease progression independent of dystrophin restoration [1]. Phase 3 trial data demonstrated statistically significant but modest slowing of functional decline compared with a placebo [11].

Common adverse events include gastrointestinal symptoms such as nausea, vomiting, diarrhea, and abdominal pain, which can be particularly problematic in non-ambulatory patients. Laboratory monitoring is required, as givinostat can also cause thrombocytopenia and hypertriglyceridemia [1]. Dosing is weight-based and administered orally twice daily [12].

Dose reduction is not uncommon and recommended for moderate to severe diarrhea, platelet counts less than 150 × 10^9^/L, and hypertriglyceridemia above 300 md/dL [1]. Electrocardiographic monitoring is advised in patients with underlying cardiac disease or concomitant medications associated with QT prolongation on an electrocardiogram (ECG) [1,12]. Laboratory monitoring includes complete blood counts every two weeks for the first two months and every three months thereafter, with triglyceride assessment at one, three, and six months and then every six months.

While givinostat represents an important addition as the first approved non-steroidal disease-modifying therapy for DMD, its long-term clinical impact, optimal patient selection, and role alongside other therapies continue to evolve [12].

### 5.3. Exon-Skipping Approach

Exon-skipping therapies aim to restore the dystrophin reading frame of the dystrophin gene by modulating pre-mRNA splicing, enabling the production of a truncated but partially functional dystrophin protein [13]. While conceptually designed to convert DMD into a less severe Becker muscular dystrophy (BMD) phenotype postnatally, currently approved exon-skipping therapies produce low levels of dystrophin, and the extent to which this translates into long-term and durable clinical benefit remains uncertain [14].

Four exon-skipping antisense oligonucleotides are currently FDA-approved for DMD: eteplirsen (exon 51 skipping, approved in 2016), golodirsen (exon 53 skipping, approved in 2019), viltolarsen (exon 53 skipping, approved in 2020), and casimersen (exon 45 skipping, approved in 2021). These are administered through weekly intravenous (IV) infusions, and they can collectively treat approximately 30% of the DMD population [15]. Truncated dystrophin restoration with these therapies ranges from approximately 1% to 6% of normal levels. For eteplirsen, the mean dystrophin production reached approximately 0.93% of the normal level after 48 weeks in initial trials [16]. Golodirsen achieved mean dystrophin levels of 1.02% (range 0.09–4.30%) of the normal level after 48 weeks [17]. Viltolarsen demonstrated the highest reported levels among approved therapies, with mean dystrophin production of 5.7% (low dose) and 5.9% at week 25, with 94% of participants achieving levels greater than 2% of the normal [18]. Casimersen showed satisfactory safety and tolerability profiles in phase 3 trials, though specific dystrophin percentages were not detailed in the approval data.

Currently approved exon-skipping therapies’ limited efficacy is suspected to largely be due to inadequate delivery to target tissues, as all approved antisense oligonucleotides (ASOs) are phosphorodiamidate morpholino oligomers (PMOs), which are uncharged and do not bind serum proteins. Consequently, PMOs exhibit low bioavailability and are rapidly cleared by the kidneys following intravenous administration. To address these challenges, multiple strategies are under active investigation and some have already been implemented to enhance tissue uptake and improve therapeutic efficacy [14]. These include peptide-conjugated morpholinos (PPMOs) for improved muscle targeting, transferrin receptor-mediated delivery platforms that exploit receptor-mediated endocytosis to enhance skeletal and cardiac muscle uptake, and novel stereochemical modifications of the 2′-O-methyl backbone designed to increase potency while reducing the required dose and adverse events [15,19].

As a result, exon-skipping therapies should be viewed as biologically active but clinically modest interventions best considered within a long-term treatment strategy rather than as standalone disease-altering therapies.

### 5.4. Mutation-Agnostic Clinical Trials

#### 5.4.1. Selective Fast Skeletal Muscle Myosin Inhibitors

Sevasemten (EDG-5506) is an oral medication that targets a key contributor to muscle damage in DMD: excessive contraction of fast muscle fibers. In dystrophin-deficient muscle, mechanical stress from contraction renders fast-twitch fibers particularly susceptible, leading to sarcolemmal injury and muscle fiber breakdown [20]. This approach is notable because it addresses the downstream consequences of dystrophin deficiency rather than attempting to restore the dystrophin protein itself, potentially offering a mutation-agnostic treatment. As of December 2025, there is no consensus on the optimal dosing for this medication. The phase 2 LYNX trial (NCT05540860) is currently evaluating sevasemten in children with DMD to determine optimal dosing, safety, and efficacy in this population. In BMD, sevasemten administration resulted in substantial reductions in serum biomarkers of muscle injury: 70% decrease in creatine kinase, 98% decrease in fast skeletal muscle troponin I, and 45% decrease in myoglobin [21].

#### 5.4.2. Satellite Cell-Modulating Therapy

Another novel, mutation-agnostic therapeutic strategy focuses on restoring the muscle’s regenerative capacity via targeting a disease-associated defect in satellite cell biology. Dystrophin deficiency disrupts asymmetric satellite cell division, leading to the progressive depletion of the muscle stem cell pool and impaired regeneration. By inhibiting AP2-associated kinase 1 (AAK1), SAT-3247, the balance of asymmetric satellite cell division can be restored, enhancing myogenic progenitor production and ultimately promoting muscle repair. In preclinical studies using mdx and other mouse models, AAK1 inhibition improved muscle regeneration, increased myofiber size, and reduced fibrosis [22]. SAT-3247 has now advanced into early clinical development, where ongoing studies are evaluating safety, pharmacokinetics, and exploratory biomarkers of muscle health (NCT07287189).

#### 5.4.3. Translational Readthrough Agent

Ataluren is an oral small molecule developed for patients with DMD caused by nonsense (premature stop-codon) mutations, which account for approximately 10–15% of cases [23,24]. Ataluren promotes the ribosomal readthrough of premature stop codons during mRNA translation, enabling the production of full-length or near-full-length dystrophin proteins without altering the underlying DNA sequence [23]. Unlike exon-skipping or gene replacement strategies, ataluren acts at the post-transcriptional level and preserves endogenous dystrophin regulation [24].

Clinical trials evaluating ataluren have demonstrated variable and modest functional benefit, with the greatest signal observed in ambulatory patients with intermediate baseline functional capacity [25]. While dystrophin expression following treatment is typically low and heterogeneous, some studies have suggested a delay in disease progression, including prolonged ambulation in select patient subgroups [26,27]. Ataluren has generally been well tolerated, with the most commonly reported adverse events including gastrointestinal symptoms and headache [28].

Despite receiving conditional approval in Europe, ataluren has not been approved in the United States, largely due to inconsistent efficacy across clinical endpoints [29,30]. As a result, its role in contemporary DMD management remains limited and somewhat controversial [25,29]. Nevertheless, ataluren represents one of the earliest examples of precision, mutation-specific therapy in DMD and highlights both the potential and the challenges of pharmacologic approaches aimed at restoring dystrophin production [23].

### 5.5. Gene Therapy

Gene therapy for DMD uses adeno-associated virus (AAV) vectors to deliver a truncated dystrophin gene (micro-dystrophin) that restores functional protein expression in the muscle. Because the full-length dystrophin gene (~14 kb) exceeds the packaging capacity of AAV vectors (~4.7 kb), these therapies use internally deleted constructs containing only the most critical functional domains, similar to the naturally occurring truncated dystrophin seen in the milder Becker muscular dystrophy [31]. Importantly, micro-dystrophins do not fully restore native dystrophin expression but functionally convert Duchenne muscular dystrophy into a Becker-like state. As such, gene therapy should be regarded as a disease-modifying intervention rather than a curative approach.

Delandistrogene moxeparvovec-rockl (Elevidys) is the only approved AAV-based micro-dystrophin gene therapy for DMD. The therapy is administered as a single-dose intravenous infusion at 1.33 × 10^14^ vg/kg for patients weighing less than 70 kg or 9.31 × 10^15^ vg for patients weighing 70 kg or more. Delandistrogene moxeparvovec-rockl initially received accelerated FDA approval in 2023 based on micro-dystrophin expression as a surrogate biomarker and subsequently received full approval in 2024 for ambulatory boys aged 4 years and older [32]. The pivotal EMBARK phase 3 trial of delandistrogene moxeparvovec-rockl (NCT05096221) did not meet its primary endpoint, showing no statistically significant improvement in the NSAA score at week 52 compared with the placebo despite robust micro-dystrophin expression at week 12 and numerical trends favoring treatment on several secondary functional endpoints, including Time to Rise, 10 m Walk/Run velocity, and time to ascend four steps [33]. Long-term follow-up from earlier phase 1/2a studies and the ENDEAVOR study suggest potential sustained benefits, with treated patients showing stabilization or improvement in NSAA scores over 1–4 years and statistically significant differences compared with external natural history controls, indicating that micro-dystrophin expression may translate into functional gains over time [34].

From a safety perspective, delandistrogene moxeparvovec-rockl is associated with treatment-related adverse events requiring medical intervention in approximately 15% of patients (13 of 85 patients across clinical trials), including vomiting, myocarditis, acute liver injury, and immune-mediated myositis. Common adverse reactions (≥5% incidence) include vomiting, nausea, liver injury, fever, and thrombocytopenia. Serious adverse events (SAEs) reported in EMBARK and earlier clinical studies included acute liver injury requiring hospitalization, immune-mediated myositis, and myocarditis, reflecting immune activation related to vector delivery and transgene expression [35]. Specifically, in the EMBARK trial, 674 adverse events were recorded in the delandistrogene moxeparvovec-rockl group compared with 514 in the placebo group, with no deaths or discontinuations due to adverse events during the 52-week study period. Seven patients (11.1%) experienced 10 treatment-related serious adverse events, which included pyrexia, nausea, vomiting, myocarditis, transient elevation of liver enzyme, hepatoxicity, liver injury, and rhabdomyolysis. The onset of these events was day 1 for myocarditis, nausea, vomiting and pyrexia; day 2 for rhabdomyolysis; days 30–51 for liver-related events; and day 92 for myositis [33].

Similar safety findings were observed in the ENDEAVOR trial, in which 18 treatment-related adverse events were reported, all occurring within 70 days post-infusion and resolving with appropriate management. In addition, two cases of immune-mediated myositis were observed approximately four weeks following infusion. These events manifested with severe muscle weakness, myalgia, dysphagia, and dyspnea. Subsequent immunologic investigations demonstrated that these reactions were driven by a T-cell-mediated immune response against specific micro-dystrophin peptides corresponding to exons 8 and 9 of the DMD gene [36]. As a result, subsequent clinical protocols were amended to exclude patients with mutations involving exon 9 (and, in some cases, exon 8 [32,36]). Two cases of acute liver failure with fatal outcomes have occurred in non-ambulatory patients treated with delandistrogene moxeparvovec-rockl, both during clinical development and in the post-marketing setting, with symptom onset typically within eight weeks of administration [37]. Non-ambulatory status appears to represent a significant risk factor for severe hepatotoxicity, as the FDA specifically highlights fatal acute liver failure in this population. Additionally, patients with pre-existing liver impairment, chronic hepatic disease, or acute liver dysfunction may be at an increased risk for serious liver injury or acute liver failure following treatment. As a result of these safety findings, since June 2025, the manufacturer has voluntarily suspended the U.S. distribution of delandistrogene moxeparvovec-rockl for the treatment of non-ambulatory DMD patients [38].

It remains uncertain how long gene therapy will persist and whether therapeutic benefits will be maintained throughout an individual’s lifespan. The American Academy of Neurology notes that therapy may not promote expression for an entire lifespan, raising important questions about redosing [34].

Following the success of delandistrogene moxeparvovec-rockl, several organizations have sought similar micro-dystrophin replacement strategies with mixed success. Gene therapy for DMD uses adeno-associated virus (AAV) vectors to deliver a truncated dystrophin gene (micro-dystrophin) that restores functional protein expression in the muscle. Because the full-length dystrophin gene (~14 kb) exceeds the packaging capacity of AAV vectors (~4.7 kb), these therapies use internally deleted constructs containing only the most critical functional domains, similar to the naturally occurring truncated dystrophin seen in the milder Becker muscular dystrophy [31]. Importantly, micro-dystrophins do not fully restore native dystrophin expression but functionally convert Duchenne muscular dystrophy into a Becker-like state. Gene therapy should therefore be viewed as a disease-modifying intervention rather than a cure, with the goal of slowing disease progression, preserving motor function, and delaying cardiopulmonary decline.

Affinity Duchenne (NCT05693142): This phase 1/2/3 open-label study evaluates a single IV infusion of RGX-202, a recombinant AAV8 vector encoding a novel micro-dystrophins transgene in males with Duchenne muscular dystrophy. Presently, the trial assesses safety, tolerability, efficacy, pharmacodynamics, and pharmacokinetics. RGX-202 is the only micro-dystrophin gene therapy in clinical development to include the C-terminal domain, which recruits α1- and β1-syntrophins and α-dystrobrevin as part of the dystrophin-associated protein complex (DAPC). Preclinical data suggest that inclusion of this evolutionarily conserved C-terminal domain results in higher micro-dystrophin levels in transduced muscles, more effective DAPC recruitment to the sarcolemma, improved histopathological outcomes, and increased muscle force and resistance to contraction-induced damage [39,40]. The trial employs a robust immunosuppression regimen to mitigate immune responses against both the AAV8 capsid and the micro-dystrophin transgene, a critical consideration given that immune-mediated toxicities including myositis, myocarditis, and hepatotoxicity have emerged as significant challenges in other AAV–micro-dystrophin programs [39].

Inspire Duchenne (NCT06138639): This phase 1/2 multicenter, open-label study investigates SGT-003, delivered as a single IV infusion using an AAV serotype SLB101 vector carrying the human micro-dystrophins gene (h-µD5) in males with DMD. The study evaluates safety, tolerability, and efficacy. The program initially faced significant setbacks when its phase I/II IGNITE DMD trial was placed on clinical hold in 2019 following serious adverse events in treated patients. After implementing enhanced safety measures and a more robust immunosuppression protocol, the trial resumed and continued enrollment [41].

Ascend (NCT06817382): This is a phase 1, multicenter study that investigates the safety and tolerability of a single dose of INS1201 via intrathecal (IT) administration in ambulatory male participants with DMD. Although still in its initial phases, it represents a promising approach, particularly for patients with pre-existing anti-AAV antibodies who may be ineligible for systemic AAV-based gene therapies.

Successful administration of AAV-based gene therapy requires careful patient selection and extensive pre-infusion evaluation. Patients with pre-existing neutralizing antibodies to the specific AAV serotype are usually excluded from clinical trials, as pre-existing immunity can prevent effective transgene delivery and increase safety risks [42,43]. Baseline cardiac and hepatic function must meet eligibility criteria, given the risks of immune-mediated myocarditis, transaminitis, and systemic inflammatory responses. Peri-infusion immunosuppression with high-dose corticosteroids, sirolimus, or a complement inhibitor is required to mitigate immune reactions to both the viral capsid and transgene expression.

Long-term durability of micro-dystrophins expression remains uncertain, particularly in the context of ongoing muscle growth and turnover in younger patients, creating the clinical conundrum of identifying the optimal age to administer treatment. As a result, extensive counseling is required to align family expectations with the known benefits, limitations, and uncertainties of gene therapy and to contextualize gene therapy as one component of longitudinal, multidisciplinary DMD care rather than a standalone solution.

Impact (NCT07160634): This is a phase 3, multicenter, double-blind, placebo-controlled study that evaluates SGT-003, an investigational gene therapy administered as a single IV infusion in ambulant males with DMD. The design investigates the safety, tolerability, and efficacy of SGT-003, which delivers a micro-dystrophin transgene via a proprietary capsid in boys with genetically confirmed DMD across specified age cohorts. Participants are randomized to receive either the active therapy or placebo, with primary endpoints focused on functional outcomes and disease progression measures. Recruitment is limited to male subjects within a defined young age range, and the trial continues to enroll globally.

Transplantation of Normal Myoblasts in Duchenne Muscular Dystrophy (NCT02196467): This phase I/II, single-center, randomized, intramuscular myoblast transplantation trial investigated the safety and preliminary efficacy of high-density injections of normal donor myoblasts into the extensor carpi radialis muscle of ambulatory patients with Duchenne muscular dystrophy (DMD). Patients received escalating volumes of 30 million donor myoblasts/cm^3^ in a stepwise fashion into the target muscle under tacrolimus immunosuppression, while the contralateral muscle was injected with saline as an internal control. The primary objective was to assess the safety and tolerability of localized myoblast delivery, with secondary endpoints including muscle strength and fatigue, comparing injected to control muscles over 12–24 weeks post-transplant; the continuation of immunosuppression to six months was planned for cases showing strength improvement. Prior phase I studies had demonstrated donor dystrophin expression in DMD myofibers following satellite cell/myoblast transplantation but with limited functional benefit, and this protocol was built on that groundwork to evaluate whether a high-density myoblast delivery with controlled immunosuppression could augment strength in a clinically meaningful way.

All the treatments described in this text are condensed in Table 1.

## 6. Clinical Scenarios

The expanding therapeutic landscape for DMD has increased the complexity of clinical decision-making. Treatment selection now requires the integration of disease stage, genotype, comorbidities, family priorities, and evolving regulatory considerations. The following clinical scenarios illustrate common decision points encountered in contemporary DMD care.

### 6.1. Clinical Scenario Number 1

A 4-year-old boy presents with a classic DMD phenotype with an exon 50 deletion, amenable to exon 51 skipping. The initial and foundational decision concerns corticosteroid therapy, which remains the backbone of disease management. Clinicians must guide families through selection among prednisone, deflazacort, or vamorolone, as well as daily versus intermittent dosing strategies. This decision balances efficacy, growth, and weight concerns, behavioral tolerability, comorbid risk factors, and family preferences informed by the experience of the treating DMD center.

Given the patient’s young age, early disease stage, and confirmed mutation, gene replacement represents the next major topic of discussion. Currently, delandistrogene moxeparvovec is the only FDA-approved gene therapy for DMD, delivering a micro-dystrophins transgene that theoretically converts DMD to a Becker-like state. However, with physician guidance, families must decide between receiving an approved product (i.e., delandistrogene moxeparvovec-rockl) versus considering exploring next-generation vectors for micro-dystrophin-replacement strategies (e.g., SGT-003, RGX-202, or INS1201). Families must be cognizant that gene therapy does not restore full-length dystrophin and does not constitute a cure. Key considerations include pre-existing AAV antibody status, baseline cardiac, pulmonary, and hepatic function, the need for high-dose peri-infusion corticosteroids, and uncertainty regarding long-term durability. As trials advance with improved promoters, optimized capsids, improved immunosuppression, and potential redosing strategies, clinicians must help families weigh the certainties of an approved therapy against the possible advantages but also risks of investigational options and uncertainty of potentially better but not guaranteed products available in the future.

Because this patient’s mutation is amenable to exon 51 skipping, exon-skipping therapy represents an additional, available disease-modifying treatment option. Eteplirsen has demonstrated modest clinical benefits but requires lifelong intravenous infusions, creating significant logistical and financial burden. Importantly, emerging exon-skipping agents, with enhanced chemistries, may offer greater dystrophin expression. Prior gene therapy does not preclude participation to receive exon-51-skipping drugs, allowing dystrophin replacement and exon skipping to be sequentially combined, reinforcing the need for longitudinal planning rather than a single intervention.

### 6.2. Clinical Scenario Number 2

A 17-year-old non-ambulatory boy with an exon 14–17 deletion presents to clinic. Present management revolves around comprehensive multidisciplinary supportive care, alongside consideration of emerging mutation-agnostic disease-modifying therapies. As tolerated, corticosteroids should continue, given the evidence of upper limb function preservation, delayed scoliosis, and cardiopulmonary stabilization [3]. In this case, givinostat may be a reasonable option, given its mutation-agnostic mechanism [44]. Alternatively, gene replacement therapy with delandistrogene moxeparvovec-rockl has limited safety and efficacy data in this population, with significant increased risks and limited benefits in non-ambulatory individuals [35]. With an exon 14–17 deletion, there are no presently available exon-skipping approaches that he would be amenable for.

Cardiac management should include a combination of the angiotensin receptor blockers family in combination with beta blockers, mineralocorticoid receptor antagonists, and SGLT2 inhibitors, as determined to be appropriate by the treating cardiologist [6]. Respiratory care should include regular cough assist support and non-invasive ventilation as deemed appropriate, with regular pulmonary function assessment to guide the timely escalation of support [12].

Orthopedic surveillance and scoliosis management, together with interventions aimed at preserving upper limb strength and function, are critical for maintaining independence and quality of life [13].

Finally, care should be grounded in a patient-centered framework, incorporating the patient’s goals related to autonomy, quality of life, and advance care planning [14].

### 6.3. Clinical Scenario Number 3

A 10-year-old ambulatory boy with a nonsense mutation in exon 17 presents for management. Not dissimilar to the other cases, corticosteroid therapy (deflazacort, prednisone, or vamorolone) remains the cornerstone of treatment. Given the nonsense mutation, ataluren may be considered where available, as it induces the ribosomal readthrough of premature nonsense mutations to partially restore dystrophin production [15]. While ataluren received conditional approval in Europe based on pre-specified subgroup analysis showing benefits in boys with ambulatory decline, it did not meet primary endpoints in pivotal trials and is not FDA-approved. Ataluren has since followed divergent regulatory paths in Europe and the United States. In the European Union, the conditional approval was subsequently not renewed after regulators concluded that confirmatory data did not sufficiently demonstrate clinical efficacy, ultimately leading to market withdrawal in 2025 following regulatory review and appeal. In contrast, while the FDA previously declined approval, a resubmission was accepted in late 2024, with ongoing review based on additional data intended to clarify ataluren’s benefit–risk profile.

Finally, micro-dystrophin gene replacement is another option. The therapy has not been extensively studied in patients with nonsense mutations specifically, but eligibility is not restricted by mutation type [45].

## 7. Conclusions

The expanding therapeutic landscape for DMD has transformed clinical care while simultaneously increasing complexity and uncertainty. Clinicians must now guide families through nuanced decisions involving therapies with varying mechanisms, modest effect sizes, evolving safety profiles, and incomplete long-term data. Optimal care requires transparent communication regarding benefits and limitations, careful longitudinal planning, and continued reliance on multidisciplinary support management. As therapeutic innovation accelerates, the role of the clinician remains central in contextualizing emerging data, aligning treatment choices with patient and family goals, and avoiding the overinterpretation of early efficacy signals.

## Figures and Tables

**Table 1:** Summary of therapeutic options for Duchenne muscular dystrophy, including mechanisms of action, indications, dosing, benefits, adverse effects, and additional clinical considerations.

Therapy	Mechanism/Target	Age/Indication	Route/Dose	Therapeutic Benefits	Adverse Effects/Monitoring	Additional Notes
**Glucocorticoids** (Prednisone/Deflazacort/Vamorolone)	Prednisone and Deflazacort: Anti-inflammatory; slows muscle degeneration. Vamorolone: Selective dissociative corticosteroid; binds GR & MR differently.	Usually start at 4–5 y and continue after loss of ambulation.	Oral. Prednisone: 0.75 mg/kg/day; Deflazacort: 0.9 mg/kg/day; Vamorolone: 6 mg/kg/day (some respond to 2 mg/kg/day); titrate for hepatic impairment or CYP3A4 inhibitors.	Prednisone and Deflazacort: Later age for loss of ambulation, preserves upper limb and respiratory function, reduces need for scoliosis surgery. Vamorolone: Improves 6MWT (~30–45 m), NSAA (~2.5–2.9 points), linear growth.	Prednisone and Deflazacort: Adrenal insufficiency, impaired growth, increased bone resorption, glucose/fat metabolism abnormalities, delayed puberty, hemoglobin A1c, bone density scanning. Hydrocortisone for adrenal support (50–100 mg depending on age). Vamorolone: Immunosuppression, decreased BMD, adrenal insufficiency, Cushing’s syndrome, hyperglycemia, behavioral/mood changes.	Daily dosing with more adverse effects; twice-weekly regimens may be effective; no clear efficacy difference between prednisone and deflazacort. Vamorolone: No prior dose reduction needed when switching from traditional corticosteroids; taper gradually for discontinuation.
**Givinostat**	Non-steroidal disease-modifying; slows muscle degeneration.	≥6 y	Oral, weight-based, twice daily.	Slows muscle degeneration.	Nausea, vomiting, diarrhea, abdominal pain; thrombocytopenia; liver enzyme increase; hypertriglyceridemia; ECG if cardiac risk, blood count every 2 weeks or the first 2 months and every 3 months after; monitor triglycerides at 1 month, 3 months, 6 months, and every 6 months thereafter.	Dose reduction if diarrhea, platelet <150 × 10^9^/L, or triglycerides >300 mg/dL; first non-steroidal approved for DMD.
**Exon-skipping (ASOs: Eteplirsen, Golodirsen, Viltolarsen, Casimersen)**	Restores dystrophin reading frame via antisense oligonucleotides; mutation-specific.	Dependent on mutation (exons 45, 51, 53).	IV administration; dose per FDA-approved regimen.	Enables production of shortened functional dystrophin.	Limited efficacy due to poor uptake in skeletal muscle and heart; rapid renal clearance.	Only targets specific mutations; ongoing research to improve tissue uptake.
**Selective Fast Skeletal Muscle Myosin Inhibitors (Sevasemten/EDG-5506)**	Reduces excessive contraction of fast muscle fibers; targets downstream effects of dystrophin deficiency.	Children with DMD (clinical trials ongoing).	Oral; optimal dose under investigation.	Potentially reduces muscle damage across all mutations.	Safety under investigation in phase 2 trial.	Does not restore dystrophin; trials ongoing (LYNX, NCT05540860).
**Satellite Cell-Modulating Therapy (SAT-3247)**	Inhibition of AP2-assocoated kinase 1 (AAK1).	Children with DMD (clinical trials ongoing).	Oral; optimal dose under investigation.	Potentially promotes muscle repair.	Safety under investigation.	Trial NCT07287189.
**Translational Readthrough Agent**	Ribosomal readthrough of premature stop codons in *DMD* mRNA.	Ambulatory children with ≥2 years with nonsense-mutation DMD.	Oral; 40 mg/kg/day in three divided doses (10/10/20 mg/kg).	Limited and variable functional benefit in select ambulatory patients; low but detectable dystrophin production; good tolerability.	GI upset, headache; generally well tolerated; no specific lab monitoring required.	Represents an early precision-medicine approach in DMD; highlights challenges of pharmacologic dystrophin restoration.
**Gene Therapy (Affinity Duchenne/Inspire Duchenne/IMPACT)**	Viral vector delivers micro-dystrophin gene.	DMD patients (clinical trial eligibility varies).	IV infusion; dosing per trial.	Potential dystrophin restoration; long-term functional benefit under investigation.	Immune response to viral vector.	Trials ongoing (Affinity Duchenne NCT05693142, Inspire Duchenne NCT06138639), IMPACT (NCT07160634).
**Gene Therapy (ASCEND)**	Gene replacement.	3 to <5 years.	IT infusion, single dose of INS1201.	Phase 1 study, benefit under investigation.	Under investigation.	Trial ID: NCT06817382.
**Gene Therapy (Transplantation of Normal Myoblasts in Duchenne Muscular Dystrophy) (NCT02196467)**	Allogeneic myoblast transplantation restoring dystrophin via fusion with host muscle fibers.	Ambulatory male patients with Duchenne muscular dystrophy; pediatric population (exact age range defined by protocol inclusion criteria).	Intramuscular injection.	Localized dystrophin expression with exploratory safety, feasibility, and muscle strength assessment.	Injection-site reactions and immunosuppression-related toxicities requiring clinical and laboratory monitoring.	Localized non-IV approach with limited scalability.

## Data Availability

No new data were created or analyzed in this study.

## Abbreviations

**6MWT**	6-min walk test
**AAV**	Adeno-associated virus
**ALT**	Alanine transaminase
**ASO**	Antisense oligonucleotide
**AST**	Aspartate aminotransferase
**BMD**	Becker muscular dystrophy
**BMI**	Bone mass index
**CK**	Creatine kinase
**CYP3A4**	Cytochrome P450 3A4
**DMD**	Duchenne muscular dystrophy
**ECG**	Electrocardiogram
**FDA**	Food and Drug Administration
**GR**	Glucocorticoid receptor
**IT**	Intrathecal
**IV**	Intravenous
**kg**	Kilograms
**mg**	Milligrams
**mRNA**	Messenger RNA
**MR**	Mineralocorticoid receptor
**NBS**	Newborn screening
**NSAA**	North star ambulatory assessment
**PMO**	Phosphorodiamidate morpholino oligomers
**PPMO**	Peptide-conjugated PMO
**RNA**	Ribonucleic acid
**RUSP**	Recommended Uniform Screening Program
